# Antibody–Drug Conjugates—A Tutorial Review

**DOI:** 10.3390/molecules26102943

**Published:** 2021-05-15

**Authors:** Stephanie Baah, Mark Laws, Khondaker Miraz Rahman

**Affiliations:** Institute of Pharmaceutical Science, School of Cancer and Pharmaceutical Sciences, King’s College London, Franklin-Wilkins Building, 150 Stamford Street, London SE1 9NH, UK; baahstephanie@hotmail.co.uk (S.B.); mark.laws@kcl.ac.uk (M.L.)

**Keywords:** antibody–drug conjugate, ADC, monoclonal antibody, linker, cytotoxic payload, tutorial review

## Abstract

Antibody–drug conjugates (ADCs) are a family of targeted therapeutic agents for the treatment of cancer. ADC development is a rapidly expanding field of research, with over 80 ADCs currently in clinical development and eleven ADCs (nine containing small-molecule payloads and two with biological toxins) approved for use by the FDA. Compared to traditional small-molecule approaches, ADCs offer enhanced targeting of cancer cells along with reduced toxic side effects, making them an attractive prospect in the field of oncology. To this end, this tutorial review aims to serve as a reference material for ADCs and give readers a comprehensive understanding of ADCs; it explores and explains each ADC component (monoclonal antibody, linker moiety and cytotoxic payload) individually, highlights several EMA- and FDA-approved ADCs by way of case studies and offers a brief future perspective on the field of ADC research.

## 1. Introduction

Cytotoxic drugs are routinely used as part of traditional chemotherapy regimens for the treatment of different cancers. While such regimens can be effective for certain types of cancer, such as testicular cancer and Hodgkin lymphoma, the non-specific action of cytotoxic drugs means that rapidly dividing healthy cells are attacked as well as cancer cells, leading to the side effects commonly associated with chemotherapy such as hair loss, sickness and tiredness [1]. The field of targeted therapy aims to find safer, more effective therapeutic agents by exploiting subtle differences between normal and cancer cells, and an example of this approach is seen in antibody–drug conjugates (ADCs). ADCs are made up of three components—a monoclonal antibody, a linker group and a cytotoxic drug—and rank amongst the most sophisticated pharmaceuticals ever developed, combining the cancer-targeting abilities of specialised antibodies with the cancer-killing abilities of cytotoxic drugs to selectively kill cancer cells [2].

**Figure 1:**
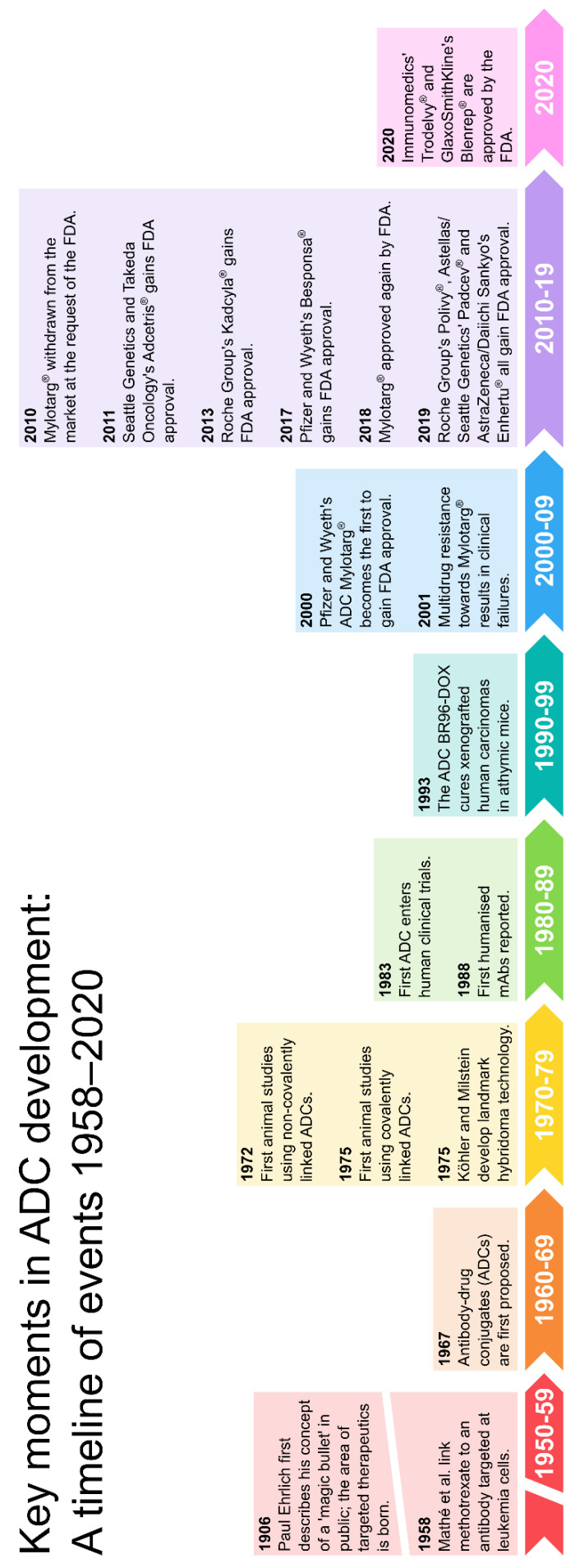
The evolution of ADC therapies. Figure redrawn based on the work of Perez et al. [7].

The German chemist Paul Ehrlich is widely credited with coining the term ‘chemotherapy’, meaning the use of chemicals to treat disease [3]. Based on his work with antibodies, Ehrlich conceived of the notion of a *zauberkugel* (“magic bullet”) that would allow the selective targeting of pathogenic microbes without harming the human body [4]. In 1983, almost eight decades later, this concept had been seized upon to achieve the first human trial of an ADC therapy [5]. Subsequent advances in antibody, linker and payload technologies have driven the development of further ADCs with improved potency and serum half-lives, reduced immunogenicity and improved specificity for cancer cells compared to earlier efforts [6]. Today, nine ADCs are approved for clinical use by the FDA (Figure 1) and the field of ADC development is rapidly expanding, promising a new generation of improved anticancer therapeutics.

## 2. Antibody–Drug Conjugate Components

### 2.1. Antibodies

#### 2.1.1. Antibody Basics

Antibodies, also known as immunoglobulins, are large, Y-shaped glycoproteins [8] that act as humanity’s bodyguards against infectious pathogens including bacteria and viruses [9]. Produced by plasma cells (immune cells that develop from activated B lymphocytes) [10], antibodies are able to selectively bind to specific antigens (structures on the outer surface of the pathogen) and therein either directly target the pathogen (by inhibiting the function of an antigen crucial to pathogen survival) or by flagging the pathogen for attack by other components of the immune system [9]. While antibodies found in the human body are polyclonal (produced by different plasma cell lineages and recognise a variety of different antigens), the antibodies commonly used in therapeutic applications are instead monoclonal (clones produced by identical copies of the same plasma cell that are specific for a single antigen) to provide a more targeted effect, with the term ‘monoclonal antibody’ commonly abbreviated to mAb [10]. This specificity for a given target antigen has driven the use of mAbs in ADCs, wherein covalent attachment of a cytotoxic agent to the mAb via a linker moiety results in delivery of the cytotoxic agent to the target cell and a reduction in toxicity compared to the cytotoxic agent alone [11].

#### 2.1.2. Antibody Structure

**Figure 2:**
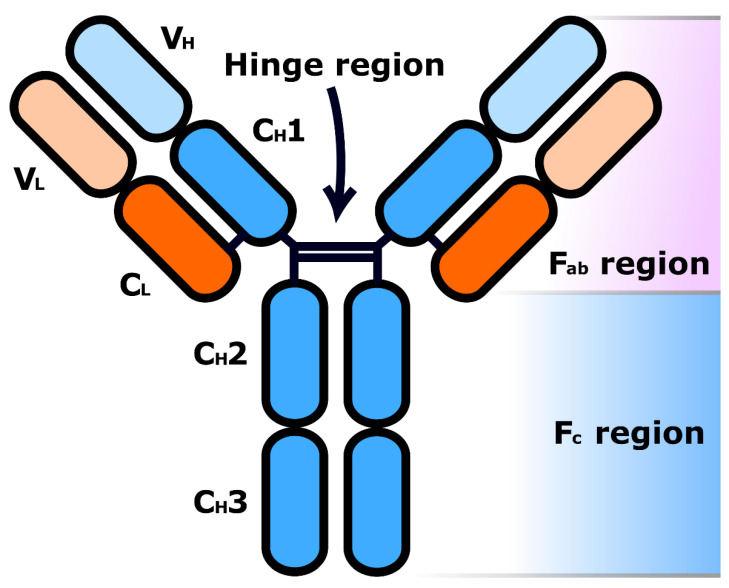
A general antibody structure (valency 2).

All antibodies share a common core structure (Figure 2) of two heavy polypeptide chains (blue) and two light chains (orange). These chains are made up of various regions, either constant (C) or variable (V) in sequence, and are assembled into a Y-shaped structure via a number of both inter- and intrachain disulphide bonds as well as various non-covalent interactions. Short carbohydrate chains attached to the heavy polypeptide chains increase the water solubility of the antibody, while a flexible “hinge” region in the middle of the antibody allows it to adjust to different arrangements of antigens on the surfaces of target cells [8].

The two fragment antigen-binding (F_ab_) regions of an antibody are each comprised of the entirety of one light chain (V_L_ and C_L_ regions) and part of one heavy chain (V_H_ and C_H_1 regions). The V_H_ and V_L_ domains form the tip of each F_ab_ region and each pair contains a paratope (antigen-binding site). Paratopes selectively bind to a specific place on a target antigen called an epitope (antigenic determinant), granting the antibody binding specificity for that antigen [10]. The location of the paratopes within variable regions of the antibody, as well as the resulting plethora of different Y-tip structures possible, explains the huge variation in antigen binding observed across different known antibodies [9]. In contrast, the fragment crystallizable (F_c_) region of the antibody, which consists of pairs of identical C_H_2 and C_H_3 regions, binds to cell surface F_c_ receptors (FcRs, found in various cell types including macrophages, B lymphocytes and natural killer cells) and allows antibodies to activate the immune system in response to a threat [11].

Antibodies found in serum can be subdivided into five isotypes (classes)—immunoglobin M (IgM), IgD, IgG, IgE and IgA-based on the amino acid sequences of their heavy chain constant regions (C_H_1, C_H_2 and C_H_3). IgM antibodies have heavy chains termed µ-chains, IgD have δ-chains, IgG have γ-chains, IgE have ε-chains and IgA have α-chains. Human antibodies also have two types of light chains termed κ- and λ-chains; antibodies can be formed of any single heavy chain type and any single light chain type [9]. Different isotypes also differ in valency (the number of “arms” the antibody has to bind to antigens with); IgM antibodies, for example, can form pentameric structures with ten paratopes each through the linking together of the F_c_ domains of five individual antibodies [12]. The IgG isotype, the most frequently used isotype for cancer immunotherapy, can be further subdivided into subclasses IgG1, IgG2, IgG3 and IgG4 based on differences including variations in amino acid sequences in the hinge and upper C_H_2 regions. These F_c_ region differences grant the subclasses differing effector functions [13].

#### 2.1.3. Antibody Function

Direct cytotoxic effects are displayed by some monoclonal antibodies used in cancer immunotherapy. For example, the humanised anti-HER2 IgG1 mAb trastuzumab (Herceptin^®^) blocks the signalling of tumour antigens associated with cell function and multiplication [14]. Regarding indirect mAb cytotoxic mechanisms, different antibody isotypes promote different types of immune responses [15]. These indirect mechanisms of action can be broadly classified as (i) antibody-dependent cellular cytotoxicity, (ii) complement-dependent cytotoxicity, (iii) complement-dependent cell-mediated cytotoxicity and (iv) promoting natural antitumour immune response mechanisms [11].

As an example of antibody-dependent cellular cytotoxicity, immune effector cells such as macrophages and natural killer cells bind to the C_H_3 region of IgG mAbs [11], triggering tumour cell death via phagocytosis (macrophages) or via the release of toxic granules (natural killer cells) [16]. In contrast, complement-dependent mechanisms instead refer to activation of the classical pathway through antigen–antibody (IgG1, IgG3, IgM) binding. The classical pathway is one of three pathways that trigger the complement system. The complement system, also known as the complement cascade, is an immunological enzymatic cascade involving over 20 plasma proteins synthesised in the liver that triggers phagocytosis, inflammation and lysis of target cells. The last effect is achieved through formation of the membrane attack complex (MAC) which forms pores in the membranes of target cells [17]. Unlike IgG1 and IgG3 which are potent activators of the classical pathway, IgG2 and IgG4 are less efficient triggers and instead induce more case-dependent, subtle responses [13]. A more detailed discussion of antibody structure and function is outside the scope of this review and can be found elsewhere [13].

#### 2.1.4. Targeting Cancer Cells with Antibodies

A key problem in using small-molecule therapeutics as anticancer chemotherapeutics is off-target toxicity, since these agents are unable to effectively discern tumour cells from normal, healthy human cells. ADCs are advantageous in this respect because the attachment of the cytotoxic agent to a tumour-targeting antibody allows more targeted treatment. To deliver the cytotoxic drug to the correct cells, the associated antibody needs to have sufficient binding affinity and specificity for its target antigen [11]. Historically, such targeting was difficult due to the inherent similarity of normal human and tumour cells—compared to, for example, bacterial cells—and the resultant lack of cell surface antigens to target [18]. However, the discovery of the first tumour-specific antigens (cell surface antigens found only on cancer cells and not on normal human cells) by Lloyd J. Old and co-workers in the late 1970s changed this, suggesting that selective targeting of tumour cells may indeed be possible [19].

When designing an ADC, the choice of target tumour-specific antigen is an important one. Common types of tumour-specific antigens include glycoproteins, extracellular matrix proteins and cell surface proteins. Some examples of tumour-specific cell surface proteins include HER2 (breast cancer), CD30 (lymphomas) and CD33 (acute myeloid leukaemia) [11]. Since approximately 90–95% of patients with acute myeloid leukaemia express the CD33 protein on the surface of their leukaemic myeloblasts, it is an example of an ideal tumour-specific target for antibody therapy [20]. It is, however, worth noting that (i) ADCs with high antigen affinity do not have high solid tumour penetration [21], (ii) the distribution of cell surface target antigen expression determines the ADC therapeutic window and (iii) a high antigen expression level in a tumour does not necessarily guarantee that an ADC will be highly effective. Polson et al. suggested that the correlation between tumour antigen density and ADC efficacy depends on the type of tumour cell [22] since the rate of internalisation of each antigen following formation of a complex with an ADC varies [6].

A potential problem with mAb-containing therapies is the effect of their large size on their pharmacokinetic properties. In ADCs, the size of mAbs relative to their payload cytotoxins means that the mAb accounts for over 90% of the mass of any given ADC. However, while this does result in reduced distribution into healthy tissue including metabolising and eliminating organs (e.g., liver, intestines, muscle, skin) [11], the leaky vasculature that characterises tumours [23] means no such problems are encountered with distribution to the tumour site [11]. In this way, mAb-based therapies enjoy both longer half-lives and greater selectivity for tumour cells [11].

#### 2.1.5. Types of Monoclonal Antibody

The four major different types of monoclonal antibodies used in antibody-based therapies are murine, chimeric, humanised and human mAbs. These are summarised in Table 1.

Historically, antisera from hyperimmunised animals has been used to treat diseases such as diphtheria. Thus, initial efforts towards achieving ADC compounds made use of mouse-derived antibodies [25]. However, subsequent identification of issues inherent to murine antibodies (high immunogenicity, poor efficacy in humans, short serum half-lives) [24], along with the discovery of hybridoma technology by Georges Köhler and Cesar Milstein in 1975 [26], led to a shift away from murine antibodies towards chimeric antibodies (antibodies with human constant regions and mouse variable regions) [24]. Hybridoma technology involves injecting a mouse with an antigen that induces an immune response and hence the release of B cells which are then fused with a myeloma (cancerous B cell) to create hybrid cell lines called hybridomas. Combining the antibody-producing ability of B cells with the increased longevity and reproductivity of the myeloma [27], hybridomas can be grown in cultures and the mAbs they produce purified [24].

However, despite the reduction in mouse-derived antibody regions, chimeric antibodies were also found to suffer from similar immunogenicity problems to murine antibodies in humans. To address this, humanised antibodies were developed in which mouse-derived regions were limited to the complementarity-determining region (CDR) loops found at the tips of the variable regions that control antigen binding specificity [25]. Finally, technological milestones including the expression of genes encoding human variable chain regions in *Escherichia coli* [28] and the expression and subsequent purification of human variable domains via phage display technology [29] have enabled the realisation of fully human antibodies (all components of the antibody are of human origin) [24].

Most ADCs currently approved for clinical use or under development utilise either humanised or human monoclonal antibodies. Humanised and human mAbs are now considered to be the first choice because their usage ensures sufficient antigen affinity and specificity, a long serum half-life and minimal immunogenicity [6]. However, exceptions to this rule do exist; brentuximab vedotin (Adcetris^®^, FDA- and EMA-approved for relapsed/refractory Hodgkin lymphoma and systemic anaplastic large cell lymphoma) is an example of an ADC that employs a chimeric mAb [30].

#### 2.1.6. Factors Influencing the Choice of Monoclonal Antibodies for ADCs

The most essential attribute of a mAb with reference to an ADC therapy is high target antigen specificity, because if an ADC forms a non-specific bond to a non-target antigen (i.e., healthy cells) then the consequences can be unpredictable and may include off-target toxicity and premature elimination from circulation due to immunogenicity, resulting in limited target exposure, decreased therapeutic effect and unwanted side effects [31]. Other important properties include high binding affinity for the target antigen, minimal immunogenicity and a half-life that enables the prerequisite target exposure for antitumour activity [32].

The IgG isotype remains the most popular with regard to ADC development, though analysis of currently approved ADC therapies (Table 2) and ADCs in clinical development reveals that this is almost exclusively limited to the IgG1 subclass. This can be explained by considering the properties of both the IgG1 subclass and the other IgG subclasses. IgG1 antibodies have similar stability in serum to both IgG2 and IgG4 antibodies (21 days) but are more potent activators of the classical complement pathway and have higher binding affinity for IgG-binding Fc-gamma receptors (FcγRs), meaning IgG1 antibodies are more effective at triggering the desired immune response [33]. IgG2 antibodies, far less effective triggers of complement, are also disfavoured due to their ability to form covalent dimers that, while thought to improve the triggering of effector functions and aid the binding of mAbs to bacterial antigens, may also cause aggregation and render the ADC ineffective [34]. IgG3 antibodies have short half-lives relative to the other IgG subclasses, meaning they are not favoured for therapeutic use despite being potent lytic agents [35]. IgG4 antibodies are also non-optimal because they can form new hybrid, bispecific mAbs (antibodies that possess non-identical paratopes in their two F_ab_ regions, resulting in the ability to bind to two different epitopes on either the same antigen or different antigens [11]) by exchanging one pair of light and heavy chains with another IgG4 antibody, though this can be overcome through replacement of the IgG4 C_H_3 domains with those of an IgG1 mAb [36]. Similar to IgG2 antibodies, they are also poor activators of effector functions [33].

However, it remains to be definitively established whether choosing a mAb according to the aforementioned criteria directly correlates with improved ADCs [11]. For example, Drachman and Senter opine that the lower immunogenicity of human mAbs versus their chimeric and humanised counterparts has yet to be proven in clinical trials since patients with advanced cancers are unable to develop the mAb-targeting antibodies previously observed in response to murine and chimeric mAb therapies [24,37]. In addition, Goldmacher and Kovtun have suggested that what was originally thought to be a positive correlation between ADC target affinity and cytotoxicity is incorrect because ADCs with high target affinity may rapidly bind to the vasculature surrounding the tumour instead of evenly across all tumour cells present [38]. It therefore remains a challenging task to design an optimised mAb for use in an ADCs.

### 2.2. Linkers

One of the biggest challenges in the development of ADCs is the selection of a suitable linker with which to conjugate the cytotoxic payload to the mAb. Linker chemistry impacts various ADC properties including toxicity, specificity, stability and potency, and thus a wide range of possible linker structures have been investigated. Linkers can be broadly classified as either cleavable (the payload is able to separate from the mAb at the tumour site) or non-cleavable (payload and mAb remain bound together, mAb is degraded following internalisation), and this, in turn, effects the modes of action of individual ADCs [39].

#### 2.2.1. Cleavable Linkers

Key to the idea of cleavable linkers in the concept of selective cleavage; the linker needs to stay intact while in the bloodstream, only cleaving to release the cytotoxic payload at the tumour site. Premature linker cleavage can result in a phenomenon called bystander killing, where the payload can diffuse into target antigen-negative cells adjacent to the tumour site and/or escape into the systemic circulation. Interestingly, while this can be disadvantageous if healthy cells are affected, bystander killing can also allow the payload to diffuse into antigen-negative tumour cells (tumour cells lacking the ADC’s target antigen) that would otherwise not be targeted by the ADC and thus aid in full tumour eradication [40]. Since crossing of cell membranes is required, bystander killing is only observed when the released payload is non-polar and charge-neutral [41].

**Figure 3:**
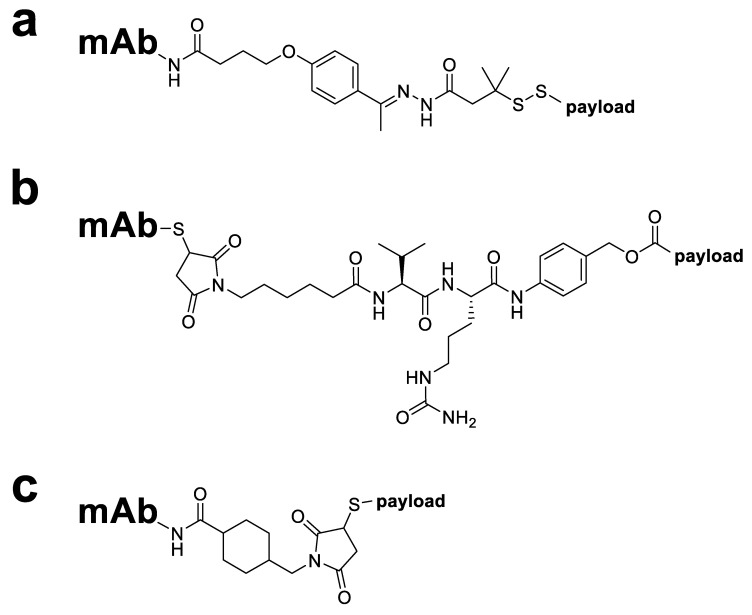
Structures of different linker groups used in ADCs. (**a**) A cleavable hydrazone linker and disulphide trigger as used in Mylotarg^®^; (**b**) an MC–Val–Cit–PABC linker, combining the non-cleavable MC and dipeptide cleavable Val–Cit linkers with a PABC spacer, as used in Adcetris^®^; (**c**) a non-cleavable MCC linker as used in Kadcyla^®^.

Selective cleavage is achieved by exploiting the unique properties of the tumour cell environment, such as the presence of certain enzymes in the cancer cell cytoplasm or a change in pH between different cellular compartments. Three types of cleavable linkers commonly used in ADCs are hydrazone, disulphide and peptide linkers (Figure 3) [11]. Hydrazone linkers, used in the commercially available ADCs Mylotarg^®^ and Besponsa^®^, are stable under the neutral pH conditions of the bloodstream but become more amenable to hydrolysis once inside the cancer cell endosomal and lysosomal compartments [42]. In contrast, disulphide linkers are reduced by glutathione to enable payload release and exploit the higher intracellular concentrations of glutathione in tumour cells (1–10 mmol/L) [43] compared with the blood (5 µmol/L) [44]. Dipeptide linkers, also known as enzyme cleavable linkers, ensure that the ADC remains stable in the circulation and only undergoes cleavage in the cancer cell intracellular lysosomal environment via lysosomal proteases, such as cathepsin B, which are overexpressed in several cancer cell types [45]. Examples include valine–citrulline (Val–Cit, found in Adcetris^®^ [30]), valine–alanine (Val–Ala, found in ADC Therapeutics’ loncastuximab tesirine [46]) and alanine–alanine (Ala–Ala, found in ImmunoGen’s IMGN632 [47]) dipeptide linkers. These are typically used alongside a *para*-aminobenzyloxycarbonyl (PABC) spacer unit which separates the dipeptide and payload moieties and allows cathepsin B better access to the cleavage site [48]. Peptide-based linkers enjoy improved stability and specificity in comparison with hydrazone and disulphide linker types due to decreased sensitivity towards serum proteases, relative pH insensitivity and selectivity for cleavage by low-pH active proteases [39].

#### 2.2.2. Non-Cleavable Linkers

While cleavable linkers undergo hydrolysis or enzymatic cleavage, non-cleavable linkers do not fragment and, following antigen-specific internalisation, only release the cytotoxic payload after complete lysosomal degradation of the mAb [6]. Key advantages of non-cleavable linkers over their cleavable competitors are that non-cleavable linkers grant ADCs longer plasma half-lives, reduced off-target toxicity and—frequently—wider therapeutic windows [39]. Common types of non-cleavable linkers used in ADCs are those based on a maleimide-type structure such as the maleimidocaproyl (MC, found in AbbVie’s depatuxizumab mafodotin [49]) and 4-maleimidomethyl cyclohexane-1-carboxylate (MCC, found in Kadcyla^®^ [49]) linkers (Figure 3) [42]. The MC linker in particular has seen varied use, employed both alone as part of a non-cleavable ADC (e.g., AbbVie’s depatuxizumab mafodotin [49]) or as a spacer unit separating a mAb and cleavable dipeptide linker sequence (e.g., Adcetris^®^ [49]), and all marketed ADCs containing monomethyl auristatin E (MMAE) or monomethyl auristatin F (MMAF) warheads use this linker [42].

#### 2.2.3. Drug–Antibody Ratio and Homogeneity

Important determinants of ADC efficacy include linker stability and ADC internalization; therefore, to achieve adequate cytotoxicity, a certain number of linker–drug units need to be attached to each mAb [11]. The average number of drug molecules per antibody is commonly referred to as the drug–antibody ratio (DAR). Hamblett et al. investigated different DARs for an MMAE payload and an anti-CD30 mAb and concluded that the optimal DAR was between two and four; increasing DAR was positively correlated with both in vitro potency and increased plasma clearance but negatively correlated with mouse maximum tolerated dose [50]. High DARs have also been linked to increased ADC aggregation [51]—undesirable since this can lead to altered ADC organ update and mechanism of clearance—and this may explain findings by Hamblett et al. that DARs of four and eight had comparable antitumour activities in vivo [50].

As well as achieving a consistent DAR, another goal for ADC design is homogeneity (consistency in the sites of attachment between individual mAbs). Both properties remain technologically challenging to achieve and it is thus unsurprising that the first clinically approved ADCs were highly heterogeneous with a wide range of different payload–linker/antibody ratios [52]. ADC homogeneity is intrinsically related to the method of conjugation of the linker to the mAb. Conjugation is typically achieved either via antibody cysteine (thiol) or lysine (ε-amino) residue side chains; while lysine side chains are often unmodified, the thiol groups of cysteine residues are almost exclusively found as disulphide bonds and therefore require selective reduction before conjugation can occur [6]. Human IgG1, for example, has 4 interchain and 12 intrachain disulphide bonds; selective reduction of the former (non-critical for the continued structural integrity of the mAb) can yield up to eight thiols for linker conjugation. In contrast, the number of lysine residues in the mAb far exceeds this, meaning that while higher DARs are possible, heterogeneity is far greater compared with cysteine-conjugated ADCs [6]. However, a case study by Yoder and co-workers assessing different modes of conjugation of a CX–DM1 linker–payload to a humanised mAb concluded that the differences between equivalent cysteine- and lysine-linked ADCs are likely to be highly case-dependent—in their case, the lysine-linked format marginally outperformed the cysteine-linked one with regard to efficacy—and they advocate the investigation of different conjugation methodologies in order to optimise any given ADC therapy [53]. The topics of ADC homogeneity and DAR consistency continue to be active areas of research and are reviewed in greater detail elsewhere [54].

#### 2.2.4. Recent Advances in Linker Technologies

**New linker classes**. Kern and colleagues investigated ADCs as a way to achieve a systemic glucocorticoid (a subclass of corticosteroid hormones that bind glucocorticoid receptors to mediate inflammation) therapy without the plethora of side effects that would be observed without mAb-directed targeting [55]. As part of this work, they reported a novel pyrophosphate diester linker that combined high plasma stability and water solubility with rapid lysosomal release via enzymatic cleavage [55]. Separately, Bargh et al. reported a pair of novel sulphatase-cleavable arylsulphate linkers with superior mouse plasma stability to Val–Ala and Val–Cit dipeptide linkers; conjugation of MMAE to trastuzumab using the arylsulphate linkers resulted in ADCs effective against HER2-positive cells [56].

**Site-specific linkers***.* As alluded to previously, ADC homogeneity can be improved by using site-specific linkers such as those that selectively append payload molecules to conserved N-glycosylated residues (amino acids with one or more sugar molecules attached via a nitrogen atom, typically the side chain amide nitrogen of asparagine) of the mAb. Faridoon and colleagues recently reported two such linkers, 2-aminobenzamidoxime and mercaptoethylpyrazolone, which they used to attach a Val–Cit–PABC–MMAE moiety to aldehyde-functionalised *N*-glycosylation sites in aqueous solution at almost neutral pH and without a catalyst [57]. Other approaches to achieving conjugation site specificity include the inclusion of genetically encoded unnatural amino acids within mAb structures, the addition of cysteine residues at predefined mAb positions for cysteine-selective attachment and tyrosine-selective click-like conjugation reactions [58].

**Tripeptide cleavable linkers***.* Valine–citrulline dipeptide linkers are stable in human plasma but less so in mouse plasma, resulting in premature payload release in mouse circulation. To circumvent this problem, Anami et al. developed a glutamic acid–valine–citrulline (EVCit) tripeptide linker which demonstrated improved stability in both mouse and human plasma while also retaining the sensitivity to proteolysis that characterised its predecessor. An anti-HER2 ADC constructed using an EVCit–PABC linker showed superior long-term stability in vivo and an improved therapeutic effect in xenograft mouse models of HER2-positive breast cancer compared to a Val–Cit-containing comparator [59]. Subsequent work by Poudel and co-workers combined the EVCit tripeptide moiety with a *meta*-amide *para*-aminobenzyl carbamate (MA–PABC) group which further enhanced mouse serum stability [60].

**Linker hydrophilicity**. Linker hydrophilicity is important; if the payload and linker are both hydrophobic, then the resulting ADCs may aggregate, leading to clearance from the blood by the reticuloendothelial system and hepatotoxicity [61]. Zhao et al. investigated whether this could be avoided in antibody–maytansinoid ADCs through use of hydrophilic linkers, such as those containing negatively charged sulfonate groups or polyethylene glycol (PEG) groups, and concluded that such linkers allow for more potent ADCs with higher DARs than achieved with traditional non-cleavable linkers while also maintaining target antigen affinity [62].

**Bioorthogonal linker cleavage**. Targeted delivery of ADC payloads has also been investigated through utilising linkers that can undergo bioorthogonal cleavage (cleavage via a chemical reaction that does not interfere with the biochemical processes of living systems). Stenton and co-workers developed a thioether propargyl carbarmate linker that selectively underwent palladium-mediated cleavage using palladium metal complex Pd(COD)Cl_2_ at room temperature. Conjugation of doxorubicin to an anti-HER2 antibody fragment via the linker yielded an ADC from which full payload release was similarly achieved in vitro. However, despite the non-toxic amounts of palladium complex required for the cleavage reaction, the toxicity of non-complexed palladium and the lack of any cell selectivity of the Pd(COD)Cl_2_ catalyst mean further work is required to make this approach viable in the clinic [63].

### 2.3. Payloads

Payloads used in ADCs tend to be cytotoxic compounds that are too toxic to be used as anticancer drugs on their own. This is because individual mAbs can accommodate relatively few payload molecules (see Drug–Antibody Ratio and Homogeneity) and only a small fraction of the administrated ADC therapy will likely reach the tumour site [11]. ADC payloads are typically 100–1000 times more potent than those used as small-molecule chemotherapeutics and have sub-nanomolar activity [64], though—since Sedlacek and co-workers estimate that less than 0.01% of an injected dose of ADC reaches each gram of a tumour [65]—cytotoxic potency in the picomolar range is not uncommon [6]. Other desirable attributes include a mechanism of action that favours toxicity against cancer cells (e.g., antimitotic agents [6]) and, if bystander killing is not wanted, the presence of ionisable functional groups (e.g., carboxylic acids) to help prevent the released cytotoxin from crossing biological membranes [41].

#### Payload Classes

**Figure 4:**
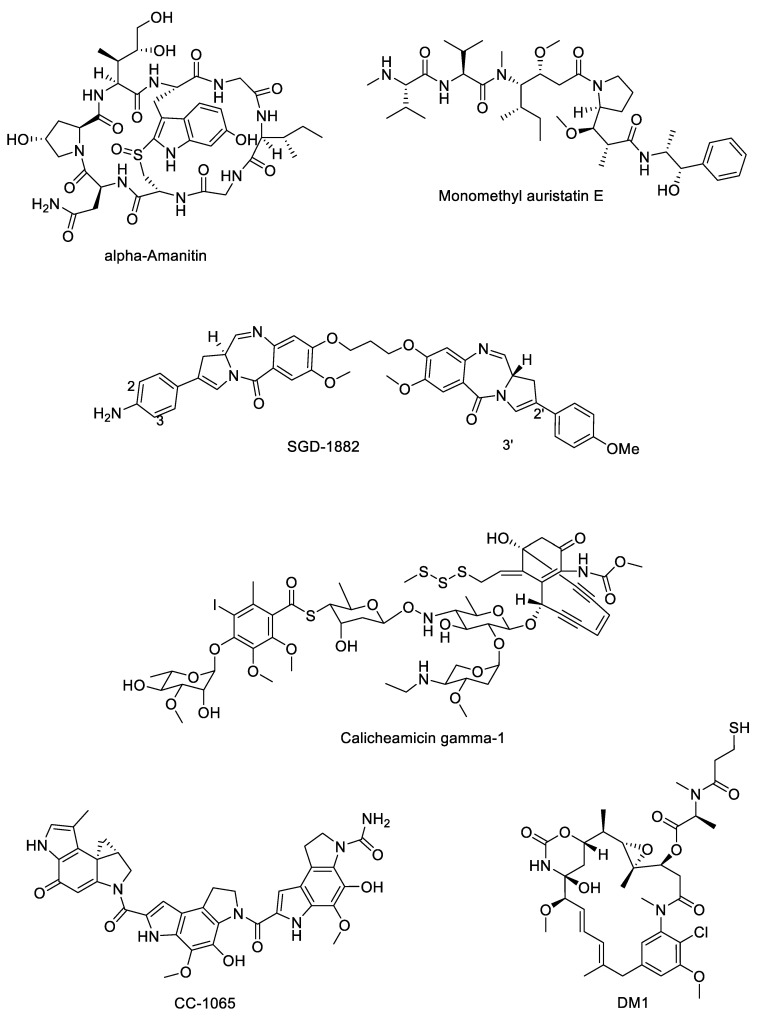
Structures of representative members of the various payload classes used in ADCs.

There are relatively few examples of payload classes available for use in ADCs, all of which are either tubulin inhibitors or DNA-interactive agents. Currently marketed ADCs contain payloads belonging to three major groups of cytotoxins; the calicheamicins (e.g., the calicheamicin γ_1_ derivative found in Mylotarg^®^), the auristatins (e.g., monomethyl auristatin E (MMAE) in Adcetris^®^) and the maytansinoids (e.g., DM1 in Kadcyla^®^). Recently, there has been interest in using additional classes of highly potent antimitotic compounds as ADC payloads such as the duocarmycins, amanitins and pyrrolobenzodiazepines [66]. Each of these classes (Figure 4) is discussed in more detail below.

**Amanitins**. The amatoxins are a group of naturally occurring bicyclic octapeptides that take their name from the genus of mushroom in which they are found (*Amanita* spp.). They bind to and inhibit the action of RNA polymerase II, disrupting transcription and protein synthesis. Of the amatoxins, α-amanitin and β-amanitin have been best studied for application as ADC payloads; attractive features of both include high potency and plasma stability, hydrophilicity, a metabolism-disrupting target, toleration of a linker moiety without loss of cytotoxicity and, most importantly, the ability to kill dormant tumour cells as well as dividing ones [67]. The most advanced ADC utilising an amanitin-type payload is Heidelberg Pharma’s HDP-101, a preclinical candidate which combines a B cell maturation antigen-targeting mAb with α-amanitin via a cleavable valine–alanine dipeptide linker [68].

**Auristatins**. The first known auristatins, dolastatins 1 and 2, were originally isolated from the wedge sea hare *Dolabella auricularia* [69]. This family of tubulin-inhibiting cytotoxins binds at the tubulin vinca alkaloid binding domain, causing cells to accumulate in metaphase arrest [70]. The structure of dolastatin 10 was used as the basis for derivatives monomethyl auristatin E (MMAE) and monomethyl auristatin F (MMAF), both of which possess an N-terminal secondary amine (rather than the tertiary amine present in dolastatin 10), allowing for straightforward linker attachment [71]. MMAF is less able to cross cell membranes than MMAE due to its C-terminal carboxylic acid group, but MMAF is also more hydrophilic, has a lesser tendency to aggregate and shows lower systemic toxicity than MMAE [72]. The marketed ADCs Adcetris^®^, Padcev^®^ and Polivy^®^ all contain MMAE payloads.

**Calicheamicins**. Originally isolated from the Gram-positive bacterium *Micromonospora echinospora* subsp. *calichensis* by scientists at Lederle Laboratories in the 1980s, the calicheamicins are a family of potent enediyne antitumour antibiotics with potent activity against Gram-positive and Gram-negative bacteria as well as human cancer cell lines [73]. These agents are DNA-interactive; upon release from the mAb, the free calicheamicin is reduced by glutathione, triggering an intramolecular Michael addition and Bergman cyclisation to form a diradical species. This binds in the minor grove of the DNA double helix, causing double-strand DNA breaks and cell death [74]. Marketed ADCs Mylotarg^®^ and Besponsa^®^ both contain calicheamicin payloads.

**Duocarmycins**. First isolated from *Streptomyces* bacteria in the 1970s, the duocarmycins are a class of minor groove-binding, DNA-alkylating natural products that form covalent bonds with the N3 positions of adenine bases and induce apoptosis [75]. First-in-class CC-1065 showed picomolar activity in leukaemia L1210 cells [76] but was hampered by hepatotoxicity [77]; its derivatives, however, have found more success. Byondis’ trastuzumab duocarmazine, a HER2-targeting ADC currently in phase 3 clinical trials, uses one such derivative, seco-DUBA, as its payload [78].

**Maytansinoids**. The cytotoxic payload used in Kadcyla^®^, mertansine (also known as DM1), is a derivative of maytansine, a natural product first extracted from the plant *Maytenus serrata* in 1972 by Kupchan and colleagues [79]. Maytansine and its derivative maytansinoids induce mitotic arrest through binding tubulin at the vinca-binding site to inhibit tubulin polymerisation [80], though maytansine itself is unsuitable for use as a chemotherapeutic due to a narrow therapeutic window [81]. Various ADCs possessing the maytansinoid payloads batansine (Bio-Thera Solutions’ BAT8001) [82], DM1 (ImmunoGen’s lorvotuzumab mertansine) [83] and DM4 (ImmunoGen’s SAR566658) [83] are currently undergoing phase 2 or phase 3 clinical evaluation.

**Pyrrolobenzodiazepines**. The pyrrolobenzodiazepine (PBD) family of DNA-interactive anticancer agents was first reported in 1965 when first-in-class anthramycin was isolated from *Streptomyces refuineus* sbsp. *thermotolerans* and characterised by Leimgruber and colleagues [84]. PBDs are minor groove-binding compounds that form a covalent bond with the C2 amino group of guanine bases through nucleophilic attack on the electrophilic imine functional group [85]. Since their discovery, various naturally occurring and synthetic analogues of anthramycin have been described including PBD monomers [86,87] and dimers (two PBDs linked together via their C8 positions) [88], and Swiss biotech firm ADC Therapeutics S.A. currently have several PBD dimer-containing ADCs (camidanlumab tesirine, loncastuximab tesirine, epratuzumab-cys-tesirine, Vadastuximab talirine) at various stages of clinical evaluation [89,90].

## 3. Antibody–Drug Conjugates in the Clinic

### 3.1. Currently Marketed Antibody–Drug Conjugates

Nine ADCs have been approved to date for clinical use [91]. They are briefly summarised in Table 2.

### 3.2. General Mode of Action

Following administration, the mAb component of the ADC recognises and binds to the cell surface antigens present on the target tumour cells. Antigen binding is followed by endocytosis, where the ADC–antigen complex is internalised within the cancer cell. Next, the payload cytotoxin needs to be released from the mAb in order to mediate cell death; in the case of non-cleavable linkers, the internalised complex is broken down via proteolysis within lysosomes, releasing the cytotoxic payload inside the cell, whereas the mechanism of payload release for ADCs with cleavable linkers varies according to the specific linker used. In all cases, the liberated payload subsequently binds to its target, leading to cell death via apoptosis [92].

### 3.3. Antibody–Drug Conjugate Case Studies

#### 3.3.1. Pfizer’s Mylotarg^®^ (Gemtuzumab Ozogamicin)

**Figure 5:**
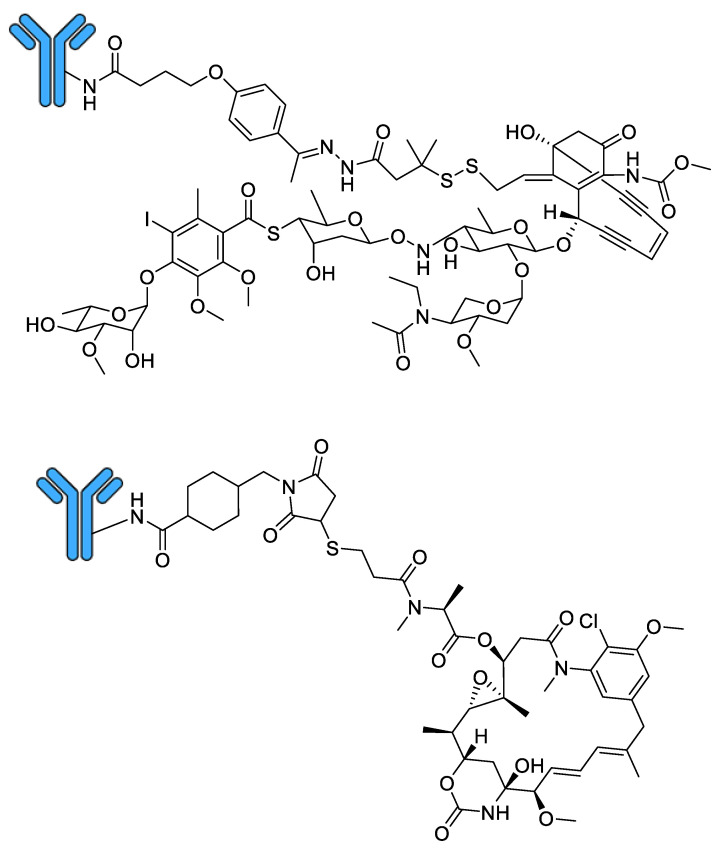
The structure of gemtuzumab ozogamicin (Mylotarg^®^, top) and trastuzumab emtansine (Kadcyla^®^, bottom).

Marketed by Pfizer Inc. as Mylotarg^®^, gemtuzumab ozogamicin comprises an anti-CD33 humanised IgG4κ monoclonal antibody connected to a calicheamicin γ_1_ derivative payload via a cleavable hydrazone linker (Figure 5) [93]. It binds preferentially to cells expressing the CD33 surface antigen, leading to internalisation of the gemtuzumab ozogamicin–CD33 complex and cleavage of the linker moiety within the low-pH environment of lysosomes via acid hydrolysis [94], whereupon the free calicheamicin is reduced by glutathione [94] and induces double-strand DNA breaks, leading to cell death [74]. Gemtuzumab ozogamicin was the first ADC to reach the clinic, approved by the FDA in 2000 under an Accelerated Approval Program for the single-agent treatment of relapsed or refractory CD33-positive AML in patients over 60 years of age unable to receive other cytotoxic chemotherapy treatments [95]. However, accelerated approval requires that post-marketing trials be conducted to confirm treatment efficacy; negative results from a number of such studies [96] (NCT00085709 and ISRCTN17161961), as well as an association with the potentially fatal condition hepatic veno-occlusive disease [97], among others, prompted Pfizer to voluntarily withdraw gemtuzumab ozogamicin from the market in 2010 [98]. However, based on the positive outcomes of subsequent trials (NCT00927498 and NCT00091234) which used fractionated dosing strategies [99], gemtuzumab ozogamicin was re-approved by the FDA in September 2017 for treatment of adults with newly diagnosed CD33-positive AML as well as for relapsed or refractory CD33-positive AML in patients over 2 years old [98].

#### 3.3.2. Genentech’s Kadcyla^®^ (Trastuzumab Emtansine)

Marketed by Genentech, Inc. as Kadcyla^®^, trastuzumab emtansine comprises an anti-HER2 humanised IgG1 monoclonal antibody connected to a DM1 payload via a non-cleavable MCC linker (Figure 5). Trastuzumab is also marketed by Genentech as a naked humanised monoclonal antibody for the treatment of HER2-positive metastatic breast cancer under the trade name Herceptin^®^ [100]. Unlike gemtuzumab ozogamicin, the non-cleavable linker present in trastuzumab emtansine means that, following entry of the ADC into the HER2-positive cancer cell via receptor-mediated endocytosis [101], mAb proteolysis inside lysosomes is needed to release the free DM1 payload [102]. Upon its release from the lysosome, DM1 binds tubulin at the vinca-binding site to inhibit tubulin polymerisation, inducing mitotic arrest and cell death [80]. Trastuzumab emtansine was approved by the FDA in February 2013 as a single-agent treatment for HER2-positive, metastatic breast cancer in patients who previously received trastuzumab and a taxane, either separately or in combination [103]; in May 2019, this was extended to include HER2-positive early breast cancer in patients with residual invasive disease after neoadjuvant taxane-based chemotherapy and trastuzumab-based treatment [104].

## 4. Conclusions and Future Perspective

With nine ADC therapies approved for clinical use by the FDA at the time of writing and at least 84 more the subjects of ongoing clinical evaluation, ADCs are clearly here to stay. This is unsurprising given the advantages in tumour cell selectivity and off-target toxicity they enjoy over conventional small-molecule chemotherapeutics. However, while the majority of ADCs discussed herein are oncology-related, the ADC concept has potential applications in a number of other, unrelated fields. This is exemplified by Genentech’s ADC DSTA4637S, an engineered IgG1 mAb conjugated to a rifamycin derivative via a cleavable valine–citrulline dipeptide linker that is currently in phase 1 trials for the treatment of MRSA infections. Lehar and colleagues reported that DSTA4637S was capable of eradicating vancomycin-resistant intracellular *S. aureus* infections and was superior to vancomycin in an *S. aureus* mouse infection model, an important discovery given the current global problem of antimicrobial resistance [105]. In the field of anti-inflammatories, Kern and co-workers sought to apply the ADC concept in order to achieve a systemic glucocorticoid medication without the side effects a purely small-molecule approach would incur [55]. We therefore look forward to further novel applications of the ADC approach both within and outside the field of oncology.

## Figures and Tables

**Table 1:** Types of monoclonal antibody [24]. *Key*: human-derived mAb regions shown in blue; mouse-derived (or for humanised mAbs, synthetic/animal-derived) mAb regions shown in orange.

Type	Suffix	Origin	Therapeutic Potential	Example	Structure
Murine	-onab	100% derived from mouse genes (both light and heavy chains)	Problems with immunogenicity, short half-lives and limited tumour site penetration	Muromonab	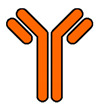
Chimeric	-ximab	35% mouse, 65% human (murine variable regions, human constant region on each chain)	Reduced immunogenicity, improved half-lives versus murine mAbs	Rituximab	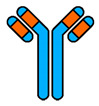
Humanised	-zumab	95% human (part of the variable domain in each chain is either synthetic or animal-derived)	Reduced immunogenicity versus chimeric mAbs	Alemtuzumab	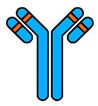
Human	-mumab	100% human (both chain types are of human origin)	Broadly reduced immunogenicity versus humanised mAbs	Adalimumab	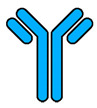

**Table 2:** ADCs currently approved for clinical use in the European Union and the USA.

ADC Name	Indication	Target Antigen	mAb	Linker	Payload	Approval Date
Brentuximab vedotin (Adcetris^®^)	Relapsed/refractory Hodgkin lymphoma, systemic anaplastic large cell lymphoma	CD30	Chimeric IgG1	Val–Cit	MMAE	25 October 2012 (EMA) 19 August 2011 (FDA)
Enfortumab vedotin (Padcev^®^)	Locally advanced/metastatic urothelial cancer	Nectin-4	Human IgG1κ	Val–Cit	MMAE	18 December 2019 (FDA)
Gemtuzumab ozogamicin (Mylotarg^®^)	Newly diagnosed, relapsed or refractory CD33-positive acute myeloid leukaemia	CD33	Humanised IgG4κ	Cleavable acid-labile hydrazone	Calicheamicin	19 April 2018 (EMA) 2 September 2017 (FDA)
Inotuzumab ozogamicin (Besponsa^®^)	Acute lymphoblastic leukaemia	CD22	Humanised IgG4	Cleavable acid-labile hydrazone	Calicheamicin	29 June 2017 (EMA) 17 August 2017 (FDA)
Polatuzumab vedotin (Polivy^®^)	Diffuse large B cell lymphoma	CD79b	Humanised IgG1	Val–Cit	MMAE	16 January 2020 (EMA) 10 June 2019 (FDA)
Sacituzumab govitecan (Trodelvy^®^)	Metastatic triple-negative breast cancer	TROP2	Humanised IgG1κ	CL2A	SN-38	22 April 2020 (FDA)
Trastuzumab deruxtecan (Enhertu^®^)	Unresectable/metastatic HER2-positive breast cancer	HER2	Humanised IgG1	Maleimide–GGFG	DXd	18 January 2021 (EMA) 20 December 2019 (FDA)
Trastuzumab emtansine (Kadcyla^®^)	Metastatic HER2-positive breast cancer	HER2	Humanised IgG1	MCC	DM1	15 November 2013 (EMA) 22 February 2013 (FDA)
Belantamab mafodotin (Blenrep^®^)	Relapsed or refractory multiple myeloma	BCMA	Humanised IgG1	MC	MMAF	25 August 2020 (EMA) 5 August 2020 (FDA)

*Abbreviations*: B cell maturation antigen (BCMA), cluster of differentiation (CD), cleavable PEG8- and triazole-containing PABC–peptide–MC linker (CL2A), derivative of maytansine (DM1), exatecan derivative (DXd), glycyn–glycyn–phenylalanyn–glycyn tetrapeptide linker (GGFG), human epidermal growth factor receptor 2 (HER2), maleimidocaproyl (MC), 4-maleimidomethyl cyclohexane-1-carboxylate (MCC), monomethyl auristatin E (MMAE), monomethyl auristatin F (MMAF), active metabolite of the topoisomerase I inhibitor irinotecan (SN-38), tumour-associated calcium signal transducer 2 (TROP2).
